# Total synthesis and structural characterization of a novel protein scaffold from the snail *Biomphalaria glabrata*

**DOI:** 10.1002/pro.70745

**Published:** 2026-08-05

**Authors:** Oleg Melnyk, Stéphanie Caby, Armelle Vigouroux, Christine Demanche, Rémi Desmet, Magalie Sénéchal, Benoît Snella, Alexandra Mougel, Céline Boidin‐Wichlacz, Aurélie Parmentier, Ugo Pasco, Sonia Cantel, Solange Moréra, Jérôme Vicogne

**Affiliations:** ^1^ Univ. Lille, CNRS, INSERM, CHU Lille Institut Pasteur de Lille, U1019 – UMR 9017 – CIIL – Center for Infection and Immunity of Lille Lille France; ^2^ Université Paris‐Saclay, CEA, CNRS Institute for Integrative Biology of the Cell (I2BC) Gif‐sur‐Yvette France; ^3^ IBMM, Univ. Montpellier ENSCM, CNRS Montpellier France

**Keywords:** *Biomphalaria glabrata*, chemical protein synthesis, disulfide‐rich proteins, miniprotein scaffold, molecular dynamics simulations, schistosomin, X‐ray crystallography

## Abstract

Disulfide‐rich miniproteins constitute compact and highly stable scaffolds of growing interest for molecular and structural engineering. Schistosomins are ~80‐residue proteins conserved across gastropods that form a long‐standing orphan family whose structure and biological roles have remained unknown. Here, we report the total chemical synthesis and structural characterization of a schistosomin isoform from *Biomphalaria glabrata*, a medically relevant intermediate host of the parasite *Schistosoma mansoni*. Using state‐of‐the‐art solid‐phase peptide synthesis, chemoselective peptide ligation, and controlled oxidative folding, we obtained homogeneous, well‐folded schistosomin suitable for biophysical and structural studies. High‐resolution X‐ray crystallography reveals a previously undescribed disulfide‐rich fold defining a new class of miniprotein scaffold. Nano differential scanning fluorimetry and circular dichroism experiments demonstrate the remarkable thermal stability of this scaffold. Complementary in silico analyses suggest that the two naturally occurring isoforms, which differ by a single residue, exhibit highly similar structural and dynamic properties. Finally, transcript and protein analyses across snail tissues provide the first spatial expression map of schistosomin in a medically relevant mollusk. Together, this work establishes schistosomin as a novel and robust miniprotein scaffold and provides a structural and biological framework for exploring its function and potential applications.

## 1 INTRODUCTION

Miniproteins are small polypeptides, typically below 10 kDa, that adopt well‐defined tertiary structures. Owing to their compact size and constrained architecture, they have emerged as attractive molecular frameworks for both functional studies and therapeutic development (Crook et al., 2020). Their structural rigidity often confers exceptional stability and binding specificity, positioning them at the interface between small molecules and large biologics. Whereas small molecules diffuse efficiently but often struggle to engage extended protein–protein interfaces, antibodies provide exquisite selectivity at the cost of limited tissue penetration and complex manufacturing. Miniproteins can, in principle, combine some of the advantages of both classes (Muttenthaler et al., 2021; Wang et al., 2022; Xiao et al., 2025; Zhang & Chen, 2022).

This growing interest has been fueled by advances in computational design (Abramson et al., 2024; Jumper et al., 2021), delivery strategies (Xiao et al., 2025) and, importantly, modern solid‐phase peptide synthesis (SPPS) (Coin et al., 2007), which in certain cases enables the synthesis of proteins comprising up to ~100 residues (Mulder et al., 2018). In parallel, the development of chemoselective peptide‐ligation methods has profoundly expanded the scope of chemical protein synthesis by allowing the selective coupling of unprotected peptide segments (Agouridas et al., 2017; Agouridas et al., 2019; Bode, 2017; Conibear et al., 2018; Dawson et al., 1994; Liu & Li, 2018; Sun et al., 2024; Zheng et al., 2013). In synergy with SPPS, these approaches enable the modular assembly of proteins bearing complex modifications, non‐canonical residues, or tailored disulfide patterns—features that often remain difficult or inaccessible through recombinant expression (Agouridas et al., 2020).

Natural ecosystems constitute a vast and still underexplored reservoir of compact, highly stabilized miniproteins. Venomous organisms such as snakes (Oliveira et al., 2022), spiders (Saez et al., 2010), and cone snails (Ahorukomeye et al., 2019; Nguyen et al., 2023; Vetter & Lewis, 2012) have yielded numerous bioactive scaffolds of pharmaceutical relevance. In contrast, miniproteins from non‐venomous mollusks remain comparatively understudied, despite the long‐standing medicinal use of terrestrial and aquatic snails (Bonnemain, 2005). Recent reports describing antimicrobial peptides from snail mucus further suggest that these organisms may harbor a broader and largely overlooked diversity of biologically active disulfide‐rich miniproteins (Rashad et al., 2025).

Among these, schistosomins form a small family of ~80‐residue, cysteine‐rich proteins initially identified in the freshwater snail *Lymnaea stagnalis* during infection with the avian trematode *Trichobilharzia ocellata* (Joosse et al., 1988; Schallig et al., 1991). Homologous sequences have since been reported in multiple gastropod species. Early studies described schistosomin as a neuroendocrine factor capable of antagonizing reproductive hormones in *L. stagnalis* (Hordijk, Schallig, et al., 1991; Hordijk, van Loenhout, et al., 1991) and highlighted its remarkable biochemical robustness, including preserved biological activity after heating at 100°C. Two schistosomin homologs were later identified in the freshwater snail *Biomphalaria glabrata*, the intermediate host of the human parasite *Schistosoma mansoni*. These two isoforms are encoded by two distinct genes but only differ by a single amino‐acid substitution (A78P) within the mature form. In *B. glabrata*, however, schistosomins' expression does not vary upon parasite infection, suggesting physiological roles beyond parasite‐induced castration (Zhang et al., 2009).

Despite more than three decades since their initial discovery, no experimental structural information has been available for any schistosomin, and their three‐dimensional architecture has remained entirely speculative. The unusual combination of small size, multiple disulfide bonds, and the absence of close homologs in protein structure databases suggested that schistosomins might adopt an as‐yet undescribed type of miniprotein fold. At the same time, the strict conservation of their cysteine framework across distantly related gastropod species raised the possibility that schistosomins could belong to a broader family of disulfide‐rich molluskan scaffolds whose structural features are encoded primarily by cysteine topology rather than primary sequence conservation. However, progress toward addressing these questions has been hampered by the low abundance of native material and the technical challenges associated with producing correctly folded protein using recombinant expression systems.

Here, we combined chemical synthesis, crystallography, biophysical approaches, and molecular dynamics simulations to characterize the structure and biochemical properties of a schistosomin isoform from *B. glabrata*. To overcome the limitations of the recombinant expression of this cysteine‐rich protein, we implemented a fully synthetic strategy based on solid‐phase peptide synthesis, chemoselective ligation of unprotected peptide segments followed by controlled oxidative folding. This approach provided homogeneous material allowing (i) its structure determination showing a new miniprotein fold and (ii) thermal stability studies using nano‐differential scanning fluorescence (nanoDSF) and circular dichroism (CD). Molecular dynamics (MD) simulations were used to evaluate differences between the two isoform scaffolds (alanine vs. proline at position 78). We also examined schistosomin transcripts and protein distribution across *B. glabrata* tissues, providing the first spatial expression map for this protein family in a medically relevant mollusk. These analyses reveal that schistosomin expression is not restricted to neuronal tissues and support a secreted, systemic distribution, challenging the view of schistosomin as a strictly neuropeptide‐like factor. Finally, structural comparisons between AlphaFold3 (AF3) (Abramson et al., 2024; Jumper et al., 2021) models and our structures indicate that schistosomin belongs to a broader family of disulfide‐rich molluskan miniproteins, including sequences tentatively classified among uncharacterized conotoxin‐like peptides (Li et al., 2025), whose three‐dimensional architecture converges despite pronounced sequence divergence.

## 2 RESULTS

### 2.1 Identification and characterization of predicted native schistosomin isoforms from *Biomphalaria glabrata*

**FIGURE 1:**
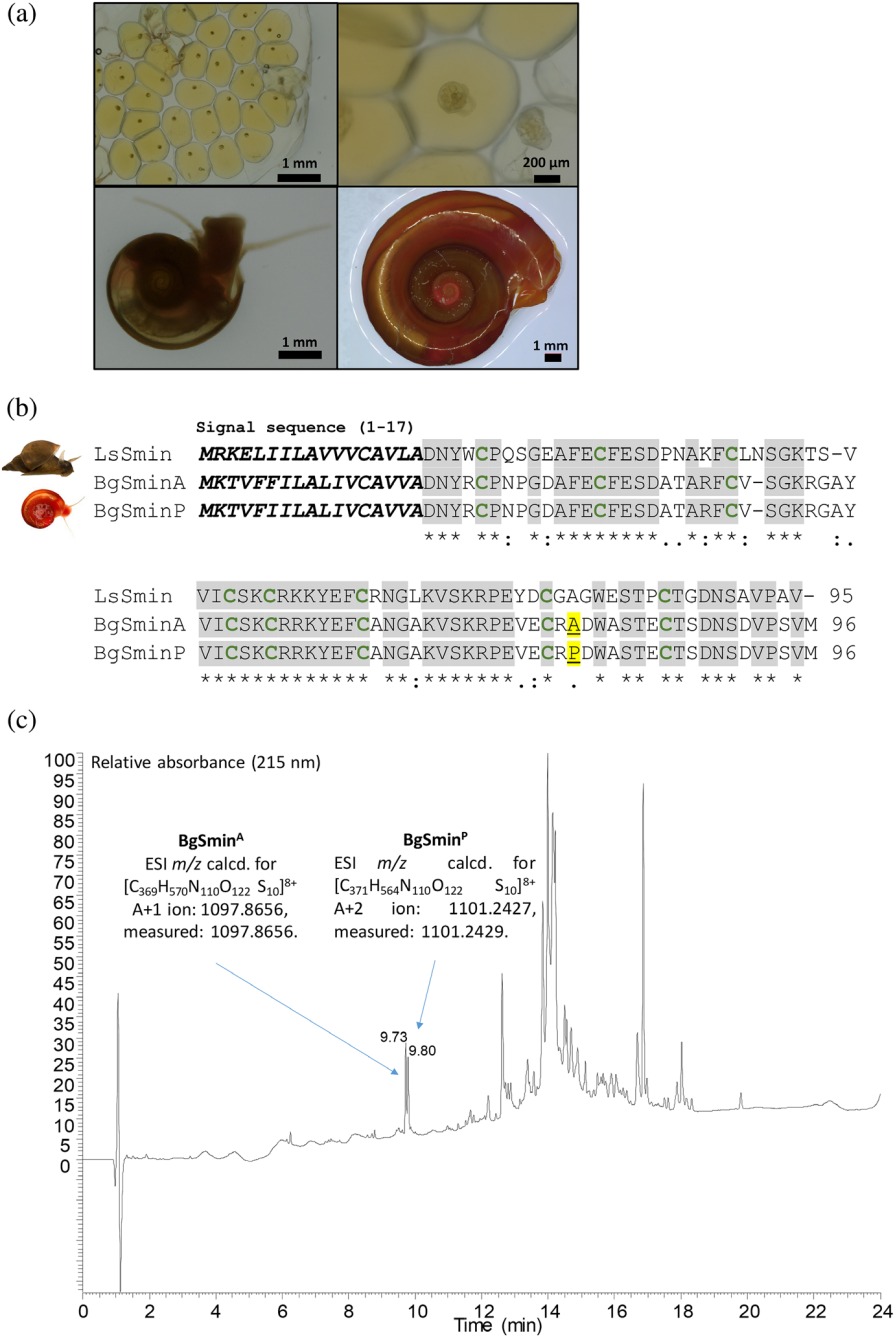
Identification of native schistosomin isoforms in *Biomphalaria glabrata*. (a) Developmental stages of *B. glabrata* providing a reproducible source of biological material for protein extraction. Freshly laid egg mass (top left), ~6‐day embryonated egg (top right), 15‐day juvenile snail (bottom left), and 3‐month adult snail (bottom right). (b) Sequence alignment of schistosomin from *B. glabrata* (BgSmin^A^ and BgSmin^P^; GenBank accession numbers EU126802 and ES488206) with schistosomin from *Lymnaea stagnalis* (LsSmin; AAB20290), the first schistosomin identified. Conserved residues are indicated by an asterisk (*), strongly similar residues by a colon (:), and weakly similar residues by a period (.). The two *B. glabrata* isoforms differ by a single amino‐acid substitution at position 78 (Ala/Pro) within the mature sequence (underlined and highlighted in yellow). (c) UPLC–MS analysis of protein extracts from adult *B. glabrata* showing two chromatographic peaks corresponding to BgSmin^A^ (9.73 min) and BgSmin^P^ (9.80 min). The HRMS assignments and measured masses of the two isoforms are indicated above the corresponding peaks. Column: ACQUITY UPLC Peptide BEH C18, 300 Å, 1.7 μm, 2.1 × 150 mm. Gradient: 0%–70% B in 15 min. Eluents: Water with 0.1% (v/v) TFA; acetonitrile with 0.1% (v/v) TFA. Flow rate: 0.4 mL min^−1^. Column temperature: 70°C.

*Biomphalaria glabrata* is readily maintained in fresh water at a temperature of 28°C under standardized lab conditions. The obtention of adult snails from the egg stage requires 3–4 months and its high fecundity provides sufficient biological material for biochemical analyses (Figure 1a). Genomic and transcriptomic data predict the expression of two schistosomin isoforms in this species (Zhang et al., 2009) (Figure 1b). These two isoforms differ only by a single amino‐acid substitution (Ala/Pro) at position 78. The two mature isoforms, which were identified in *B. glabrata* protein extracts, start at Asp18 following signal peptide cleavage as predicted, consistent with a secreted protein as observed with the *L. stagnalis* schistosomin.

Native schistosomins were isolated as two chromatographic peaks by reversed‐phase HPLC (RP‐HPLC) from protein extracts prepared from dissected foot tissues of adult snails following ammonium sulfate precipitation (Figure S1a,b). High‐resolution mass spectrometry (HRMS) analyses matched precisely the theoretical masses for the expected mature sequences minus 8 mass units, consistent with two distinct forms stabilized by four intramolecular disulfide bonds (Figures 1c and S1c,d). The two proteins were detected in comparable amounts in the extracts and are hereafter referred to as BgSmin^A^ and BgSmin^P^.

Although these analyses establish the identity of the two native schistosomin isoforms, the limited amount of material obtainable from snail tissues precludes extensive biophysical and structural characterization therefore, motivating the implementation of a total chemical synthesis strategy to obtain sufficient quantities of homogeneous material for detailed analysis.

### 2.2 Total chemical synthesis of schistosomin enables access to homogeneous and correctly folded protein

Previous attempts to produce BgSmin^A^ isoform using classical recombinant expression strategies resulted in insoluble material (Hordijk, van Loenhout, et al., 1991; Zhang et al., 2009), most likely due to incorrect disulfide bond formation. In our hands, we also obtained a heterogeneous product that was difficult to purify. These limitations led us to implement a total chemical synthesis strategy to obtain sufficient quantities of homogeneous material with full control over folding.

**FIGURE 2:**
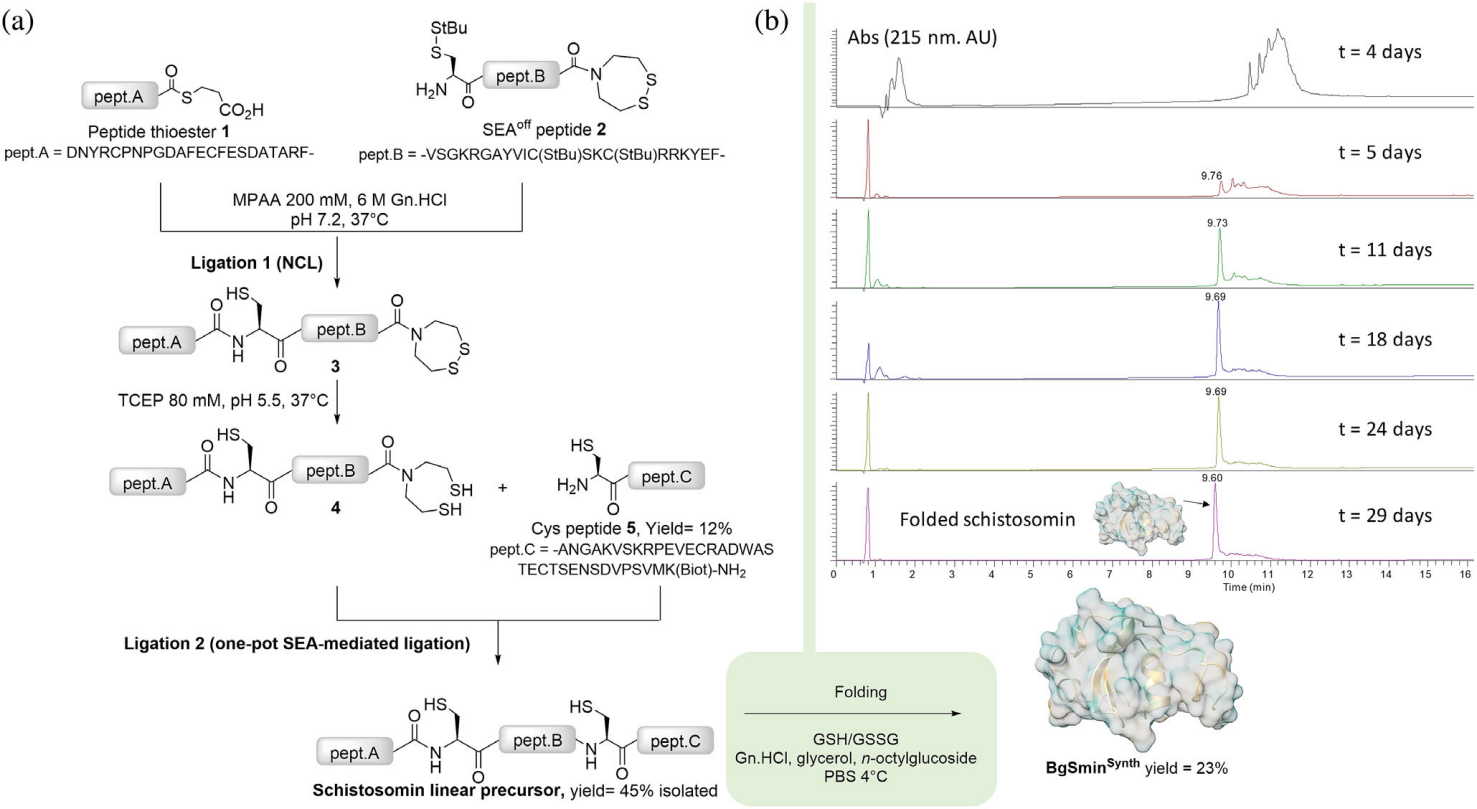
Total chemical synthesis and oxidative folding of BgSmin^Synth^. (a) Design and workflow of the chemical synthesis of BgSmin^Synth^. The mature schistosomin sequence (residues 18–96) was assembled from three peptide segments (A, B and C) produced by solid‐phase peptide synthesis (SPPS). The first ligation was achieved by native chemical ligation (NCL) between segments A and B. The resulting intermediate was then subjected to reductive activation of the SEA group to generate an in situ thioester, enabling a second SEA‐mediated ligation with segment C to afford the full‐length linear precursor. To prevent aspartimide formation during synthesis, Asp88 was substituted by Glu. In addition, a C‐terminal lysine bearing a biotin moiety (Lys97) was introduced to facilitate downstream functional studies. (b) Monitoring of oxidative folding of the synthetic BgSmin precursor by analytical UPLC. The unfolded linear precursor was first solubilized in 6 M guanidinium hydrochloride and diluted into a glutathione‐based redox buffer supplemented with glycerol and the non‐ionic detergent *n*‐octylglucoside. Folding was carried out at 4°C and progressively converged over 29 days toward a single dominant species corresponding to the correctly folded BgSmin^Synth^ protein.

The first attempts to chemically produce BgSmin^A^ isoform revealed that the Asp88‐Asn89 diad was prone to aspartimide formation. To avoid this side reaction, Asp88 was substituted by Glu. In addition, a C‐terminal lysine (Lys97) bearing a biotin moiety was introduced to facilitate future functional studies without interfering with the structural core. The synthetic approach relied on the assembly in solution of three peptide segments produced by SPPS (see detailed strategy in Figure S2). The full‐length linear precursor was obtained in only two steps through a redox‐controlled sequential native chemical ligation/bis(2‐sulfanylethyl)amido (SEA)‐mediated peptide ligation (Ollivier et al., 2010; Ollivier et al., 2012) strategy (Dawson et al., 1994) (residues 18–96; Figure 2a) and characterized by UPLC‐MS (Figure S3). The purified linear precursor was used to generate polyclonal antibodies that proved highly specific and enabled subsequent detection of native schistosomin in tissue extracts.

Folding of the synthetic schistosomin required carefully optimized oxidative conditions due to limited solubility and aggregation tendencies. A robust protocol was established by first solubilizing the linear precursor in 6 M guanidinium hydrochloride (Gn.HCl) and adding this solution to a glutathione‐based redox buffer supplemented with glycerol and non‐ionic detergent *n*‐octylglucoside. The folding was performed at low temperature (4°C), which proved to be also critical for minimizing protein aggregation. Although the oxidative folding process proceeded slowly (up to 4 weeks), it reproducibly converged toward a single dominant species, consistent with a well‐defined and thermodynamically favored disulfide connectivity (Figure 2b). UPLC–MS HRMS analyses confirmed the formation of the fully oxidized protein with the expected molecular mass (Figures S4 and S5). An additional independent synthesis and folding preparation yielded folded BgSmin^Synth^ with a final isolated yield of 17.4%, comparable to the 23% yield obtained for the initial preparation, supporting the batch‐to‐batch reproducibility of the procedure (Figure S6).

The availability of milligram quantities of homogeneous synthetic protein (BgSmin^Synth^) provided the basis for detailed structural and biophysical investigations.

### 2.3 Crystal structures of BgSmin protein reveal a novel disulfide‐rich fold

The same crystallization condition (Table 1) of BgSmin^Synth^ protein (residues 18–96) led to two different space groups (P2_1_ and C2), reflecting distinct packing arrangements that involve the additional C‐terminal biotinylated lysine residue at position 97. In both crystal forms (PDB entries 9RT6 and 9FDO; Table 1), two molecules are present in the asymmetric unit, yielding four independent and identical molecules, as indicated by the average root mean square deviation (RMSD) of 0.55 Å for all Cα atoms. In line with the very low sequence identity of BgSmin (below 10%) against the Protein Data Bank members, no homologous model could be used as a search model for molecular replacement. A reliable predicted model generated using AlphaFold2 allowed structure determination.

**TABLE 1:** Crystallographic data and refinement parameters for BgSmin^Synth^.

PDB code	9RT6 (9FDM)	9FDO
Crystallization conditions	2 M AS/ 0.1 M Tris–HCl pH 8/ 0.2 M NaBr	
Space group Cell parameters (Å, °)	P2_1_	C2
	*a* = 21.8	*a* = 87.7
	*b* = 38.4	*b* = 38.0
	*c* = 87.8	*c* = 43.2
	*β* = 91.2	*β* = 95.3
Resolution (Å)	19.7–1.9	43.6–2.07
	(2–1.9)	(2.17–2.07)
Estimated resolution limit (Å) STARANISO	1.80; 1.98; 1.94	2.12; 2.06; 2.05
No. of observed reflections	77,793 (3325)	43,741 (856)
No. of unique reflections	10,239 (512)	7585 (379)
Completeness (%)	87.8 (30.3)	84.5 (29.4)
*R*_merge_ (%)	26 (133.6)	13.3 (115.8)
*R*_pim_ (%)	10 (56.6)	5.8 (94.6)
*I*/*σ*(*I*)	5.6 (1.4)	7.7 (0.8)
CC_1/2_	0.985 (0.483)	0.994 (0.313)
*R*_work_ (%)	20.7	22.9
*R*_free_ (%)	25	27.5
rms bond deviation (Å)	0.008	0.008
rms angle deviation (°)	0.96	0.93
Average B (Å^2^)		
Protein A/B	18.7/27	33.5/35.5
Solvent	25.7	35.2
Clashscore^a^	1.68	0.82
MolProbity score	1.46	1.51
Ramachandran plot^a^ (%)		
Favored	94.00	94.16
Outliers	1.33	0.65

*Note*: Values for the highest resolution shell are in parentheses. CC_1/2_ = percentage of correlation between intensities from random hall‐dataset. STARANISO applies ellipsoidal mask: estimated resolution limits along the three crystallographic directions a*, b*, c*.

^a^ Calculated with MolProbity.

**FIGURE 3:**
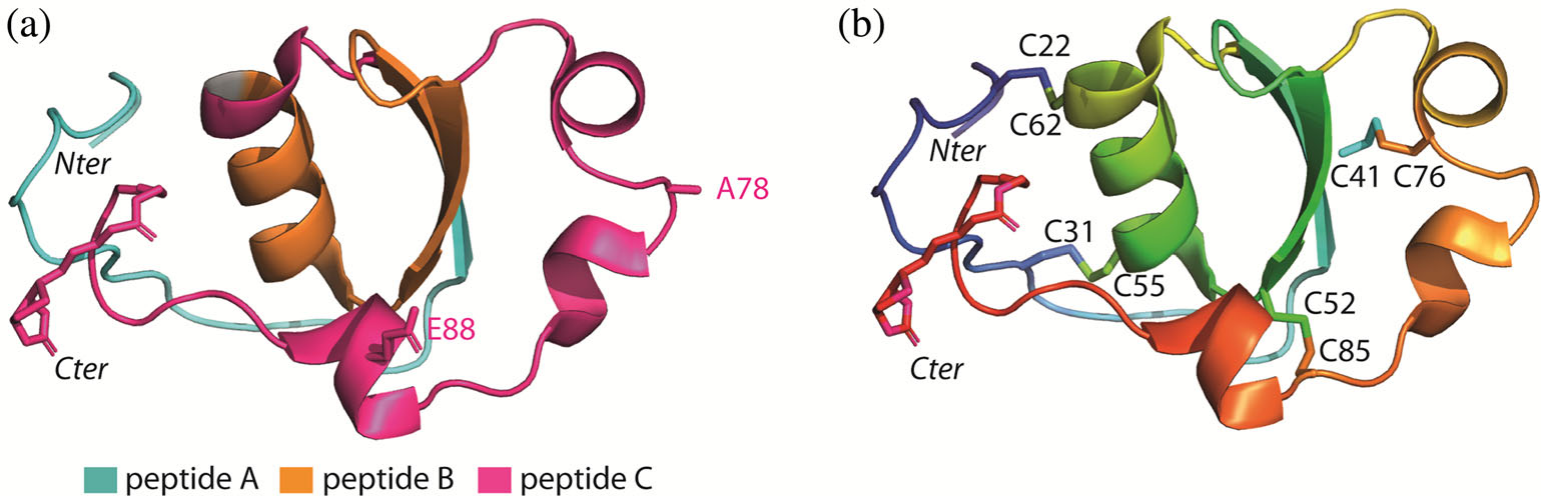
Structure of BgSmin^Synth^. (a) Ribbon representation of BgSmin^Synth^ (PDB 9FDO) colored according to the three peptide segments used for the chemical synthesis: peptide A in cyan, peptide B in orange, and peptide C in magenta. Both the N‐ and C‐termini are labeled. The C‐terminal biotinylated lysine (Lys97) is shown as sticks. The side chains of Ala78 and Glu88 are shown as sticks and labeled. (b) Ribbon representation of BgSmin^Synth^ colored from the N‐terminus in blue to the C‐terminus in red. The four intramolecular disulfide bonds, Cys22‐Cys62, Cys31‐Cys55, Cys41‐Cys76, and Cys52‐Cys85, are shown as sticks, with the corresponding cysteine residues labeled.

BgSmin^Synth^ adopts a compact monomeric fold composed of the three peptide segments used for the chemical synthesis (Figure 3a). The structure is stabilized by four intramolecular disulfide bridges (Cys22‐Cys62, Cys31‐Cys55, Cys41‐Cys76, and Cys52‐Cys85; Figure 3b), corresponding to a 1–6, 2–5, 3–7, and 4–8 connectivity pattern with no equivalent in the Protein Data Bank (PDB). The first two disulfide bonds tightly anchor the N‐terminal region (residues 18–38) to the central α‐helix spanning residues 54–64, while the latter two constrain the β‐strand elements preceding the helix (residues 39–44 and 47–52) against the C‐terminal region. As classically observed in protein structures, the two to three‐first N‐terminal residues (Asp18, Asn19, and Tyr20) are poorly defined or not visible in the electron density maps in the four molecules suggesting conformational flexibility in this region. The D88E substitution is conservative, whereas the C‐terminal Lys(Biot)‐NH_2_ extension lies outside the conserved structural core; importantly, neither of these two modifications can alter the cysteine framework that defines the schistosomin fold and disulfide‐bond connectivity.

Unlike the full‐length sequence, which includes the signal sequence, the AlphaFold3 predicted models of the mature sequence of both native BgSmin sequences (residues 18–96) resemble the crystal structures of BgSmin^Synth^, with an average RMSD value of 0.6 Å comparable to those observed between the independent crystal molecules. This predictive accuracy supports the notion that schistosomin adopts a highly constrained and energetically well‐defined fold, strongly encoded by its disulfide framework, and suggests that reliable structural models can be obtained for related schistosomin‐like sequences. Retrospectively, these observations validate the folding strategy developed for the synthetic protein. Together with the masses of native schistosomins, which are consistent with the formation of four disulfide bonds, these observations support the use of BgSmin^Synth^ as a faithful structural surrogate of native BgSmin^A^.

Given the compact architecture and dense disulfide connectivity revealed by crystallography, we next investigated the thermal stability of the schistosomin scaffold in solution using complementary biophysical approaches.

### 2.4 NanoDSF and CD analysis supports the exceptional stability of the schistosomin fold

**FIGURE 4:**
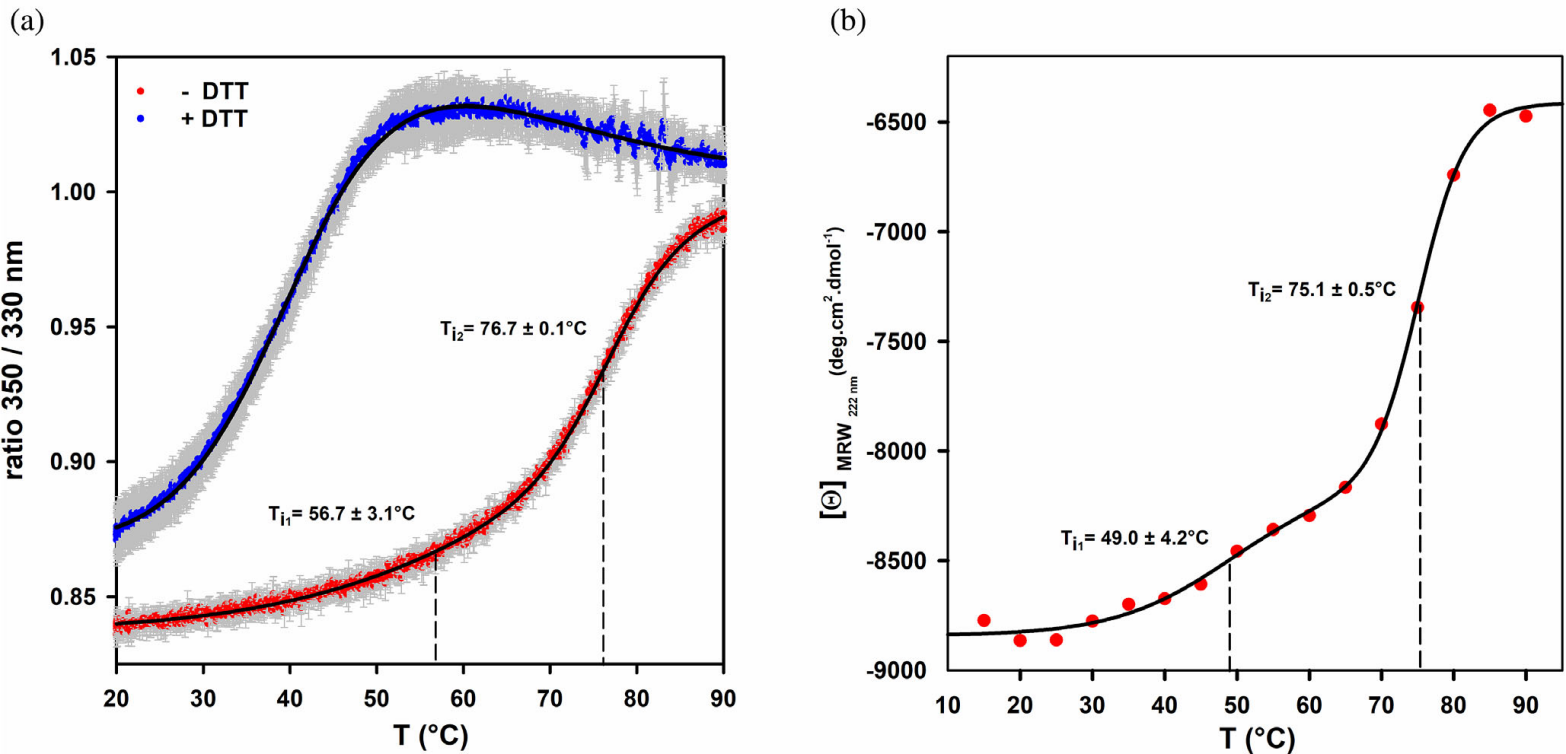
Biophysical analysis of the thermal stability of BgSmin^Synth^. (a) Thermal denaturation profile of BgSmin^Synth^ monitored by nano differential scanning fluorimetry (nanoDSF) in the absence (−DTT) or presence (+DTT) of 1 mM DTT. Measurements were performed on a Prometheus NT.48 instrument (NanoTemper Technologies) using 5 μL of BgSmin^Synth^ at 30 μM in 10 mM sodium phosphate buffer, pH 7.4, with a heating rate of 2.5°C·min^−1^. For reducing conditions, BgSmin^Synth^ was incubated with 1 mM DTT for 20 min on ice before loading into nanoDSF capillaries. The intrinsic fluorescence ratio (350/330 nm) was plotted as a function of temperature. The colored traces represent raw, unsmoothed data. Three technical replicates were performed in the absence of DTT and four technical replicates in the presence of DTT, using aliquots from the same protein preparation. Error bars represent the standard deviation (SD), and solid black lines represent the fitted curves. Apparent infection temperatures were Ti_1_ = 56.7 ± 3.1°C and Ti_2_ = 76.7 ± 0.1°C in the absence of DTT. DTT induced a marked destabilization of BgSmin^Synth^, but no reliable apparent inflection temperature could be determined under these experimental conditions. Thermal transitions were determined by fitting the data using a double‐sigmoidal regression model. (b) Thermal denaturation monitored by circular dichroism (CD). Mean residue ellipticity ([Θ]MRW) at 222 nm was plotted as a function of temperature between 15 and 90°C, using values extracted from full CD spectra recorded at 5°C intervals (Figure S7). Apparent midpoint temperatures were Ti_1_ = 49.0 ± 4.2°C and Ti_2_ = 75.1 ± 0.5°C. Thermal transitions were determined by fitting the data using a double‐sigmoidal regression model.

We first monitored temperature‐induced conformational changes using nano‐differential scanning fluorimetry (nanoDSF), by recording the intrinsic tryptophan fluorescence ratio (350/330 nm) as a function of temperature (20–90°C) from the single tryptophan (Trp80) present in the BgSmin^Synth^. The fluorescence ratio obtained with a newly prepared batch of BgSmin^Synth^ displayed a biphasic temperature dependence, indicative of two apparent unfolding transitions (Figure 4a). Fitting the data using a double‐sigmoidal model significantly improved the quality of the fit compared to a single‐transition model, as evidenced by the analysis of residuals. The two apparent inflection temperatures were Ti_1_ = 56.7 ± 3.1°C and Ti_2_ = 76.7 ± 0.12°C. Notably, the second transition was sharp and highly reproducible across replicates, in contrast to the first transition.

Under reducing conditions, DTT‐treated BgSmin^Synth^ displayed a markedly altered nanoDSF profile and a higher initial fluorescence ratio at 20°C, consistent with substantial destabilization already present at the beginning of the temperature ramp (Figure 4a). Consequently, no reliable Ti value could be assigned under reducing conditions.

In parallel, we performed complementary circular dichroism (CD) experiments by monitoring full spectral CD scans recorded between 185 and 260 nm over a temperature range of 15–90°C. These scans confirmed a progressive loss of secondary structural features upon heating (Figure S7). Analysis of the mean residue ellipticity ([Θ]_MRW_) at 222 nm revealed a similar biphasic behavior, with a major apparent inflection temperature (Ti_2_) of 75.1 ± 0.5°C (Figure 4b), whereas the lower‐temperature transition (Ti_1_ = 49.0 ± 4.2°C) appeared broader and less well‐defined. CD measurements under reducing conditions could not be reliably performed because DTT interfered with far‐UV CD measurements under our conditions.

The similar high‐temperature transition values, measured by NanoDSF and CD measurements, are consistent with the compact and stable architecture of schistosomin and likely reflect global protein unfolding. In contrast, the lower‐temperature transition, which differs between NanoDSF and CD, may reflect partial protein destabilization. The marked effect of DTT further supports an important contribution of the conserved disulfide‐bond network to the thermal stability of schistosomin.

### 2.5 Molecular dynamics simulations of A78P analog

To explore the potential impact of the amino acid residue present at position 78 (Ala versus Pro) on the conformation of BgSmin, we performed MD simulations of BgSmin^A^ D88E and BgSmin^P^ D88E at 300 K at an ionic strength corresponding to those of the hemolymph of the snail (*I* = 70 mM NaCl). MD trajectories between the A and P isoforms revealed no significant differences in overall conformational behavior. Root Mean Square Fluctuation (RMSF) profiles were nearly indistinguishable between BgSmin^A^ and BgSmin^P^ (Figure S8). Local conformational analyses indicated that A78 can adopt Φ and Ψ angles distribution that are well tolerated by proline residue, in line with (i) the structure‐based proline design tool (Proscan web server: https://proscan.ibbr.umd.edu) (Felbinger et al., 2024), which identified position 78 as highly permissive to proline substitution when applied to the schistosomin crystal structure (PDB 9FDO, chain A) and (ii) the identical AF3 overall models between BgSmin^A^ and BgSmin^P^. Therefore, A78P variation represents a conservative substitution that preserves both the structural integrity and the dynamic properties of the schistosomin scaffold.

### 2.6 Structural convergence among schistosomin‐like sequences and relationship to conotoxin disulfide‐rich peptides

To verify our hypothesis that schistosomin fold is strongly encoded by its disulfide framework, and that reliable structural models can be obtained for related schistosomin‐like sequences, we compiled, analyzed and run AF3 on all currently available sequences identified across molluskan species, which are so far restricted to freshwater and marine gastropods. A substantial fraction of these sequences originates from ConoServer (https://www.conoserver.org/), a curated database dedicated to cone snail peptides, reflecting the long‐standing focus on venom‐derived miniproteins (Kaas et al., 2012). As a consequence, cone snail sequences are overrepresented in current datasets, a bias that likely reflects uneven sampling rather than a true biological restriction of this scaffold to venomous lineages (Li et al., 2025).

**FIGURE 5:**
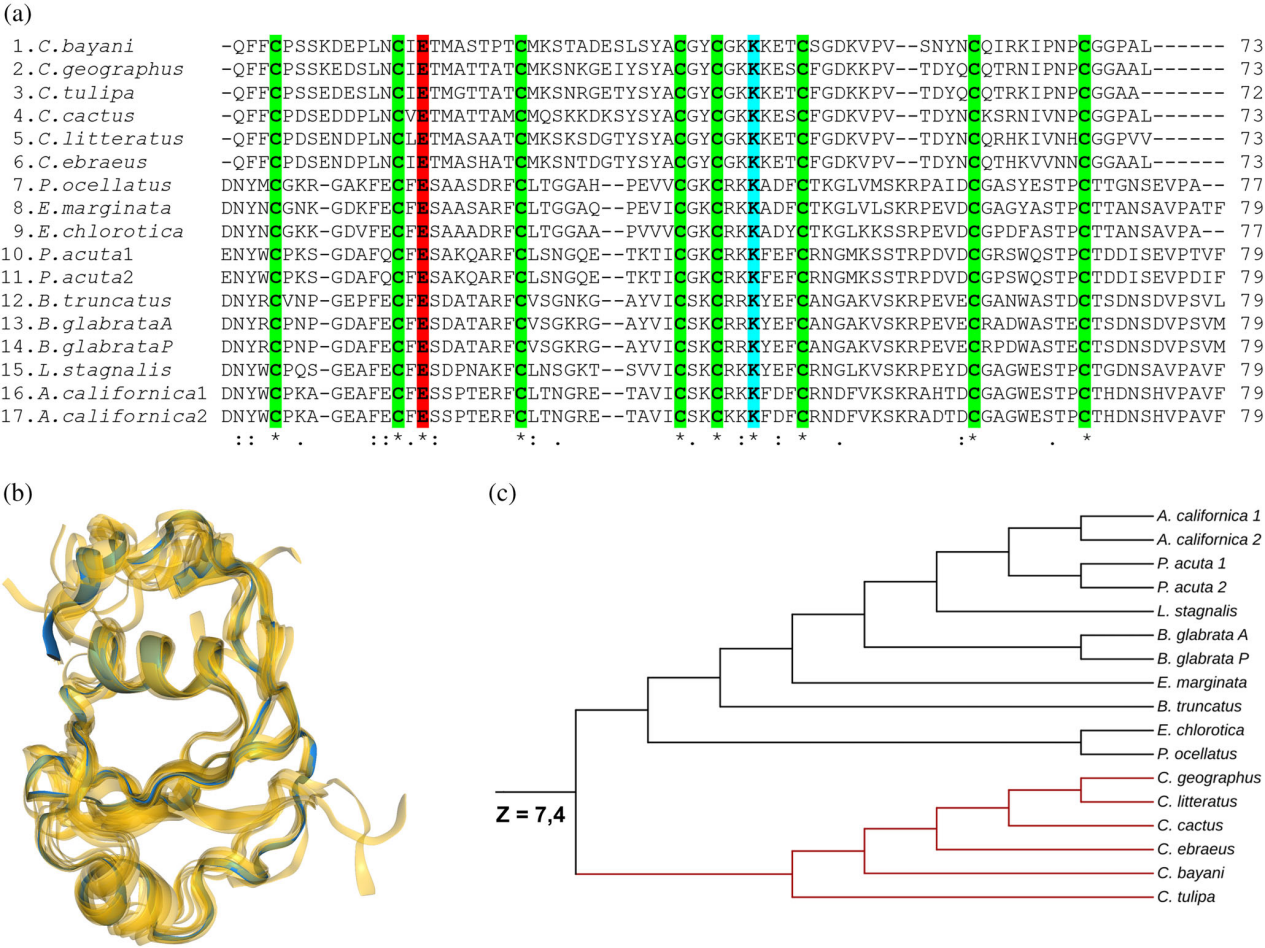
Sequence and structural comparisons of schistosomin‐like peptides across mollusks (a) Sequence alignment of schistosomin‐like peptides. Multiple sequence alignment of 17 schistosomin‐like proteins identified across gastropod species, including freshwater snails (*Lymnaea stagnalis*, *Biomphalaria glabrata*, *Bulinus truncatus* and *Physella acuta*), sea slugs (*Aplysia californica*, *Elysia marginata*, *Elysia chlorotica*, and *Plakobranchus ocellatus*), and marine cone snails (*Conus* spp.). Sequences correspond to predicted mature peptides after removal of signal peptides. Conserved cysteine residues are highlighted in green and define a characteristic disulfide framework shared across all sequences. Conserved residues are indicated by an asterisk (*), strongly similar residues by a colon (:), and weakly similar residues by a period (.). (b) Structural comparison of schistosomin‐like peptides. Superposition of AlphaFold3‐predicted structures of the 17 schistosomin‐like peptides using Foldseek. Despite low sequence identity, all structures display a highly similar compact fold stabilized by four conserved disulfide bonds, illustrating strong structural conservation across gastropod lineages, including peptides currently classified as class XXII conotoxins. (c) Structural similarity dendrogram of schistosomin‐like peptides based on DALI Z‐scores (high *Z*‐scores indicate strong structural similarity). The dendrogram reveals two main structural clusters, with a *Z*‐score threshold of 7.4 separating *Conus*‐derived peptides from heterobranch gastropod schistosomins. Branches corresponding to *Conus*‐derived peptides are shown in red.

Sequence analyses reveal that the 17 confident schistosomin‐like protein sequences identified so far, corresponding to the full‐length mature peptides after removal of the predicted signal peptides, share a strictly conserved cysteine framework despite overall sequence identities ranging from 18.6% to 98.7% (Figure 5a). Among these, only the schistosomins from *B. glabrata* and *L. stagnalis* have been experimentally characterized, while the remaining sequences are inferred from transcriptomic data. This conserved cysteine pattern therefore constitutes a robust molecular signature for identifying novel Smin‐like peptides in omics datasets. AF3 models were generated (Figure S9) and structural superposition of the 17 predicted models using Foldseek (van Kempen et al., 2024) showed a common compact three‐dimensional architecture similar to our BgSmin^Synth^ structure (Figure 5b). The DALI algorithm (DALI server: http://ekhidna2.biocenter.helsinki.fi/dali/) (Holm et al., 2023) produced a structural similarity dendrogram for these 17 predicted models, which showed the presence of distinct but closely related clusters (Figure 5c and Table S1). In particular, peptides from *Conus* species (*Caenogastropoda*) tend to group separately from schistosomins of *Heterobranchia* gastropods such as *Biomphalaria, Bulinus*, and *Lymnaea*, while remaining within the same overall structural family.

### 2.7 Schistosomin expression in the *B. glabrata* organs

**FIGURE 6:**
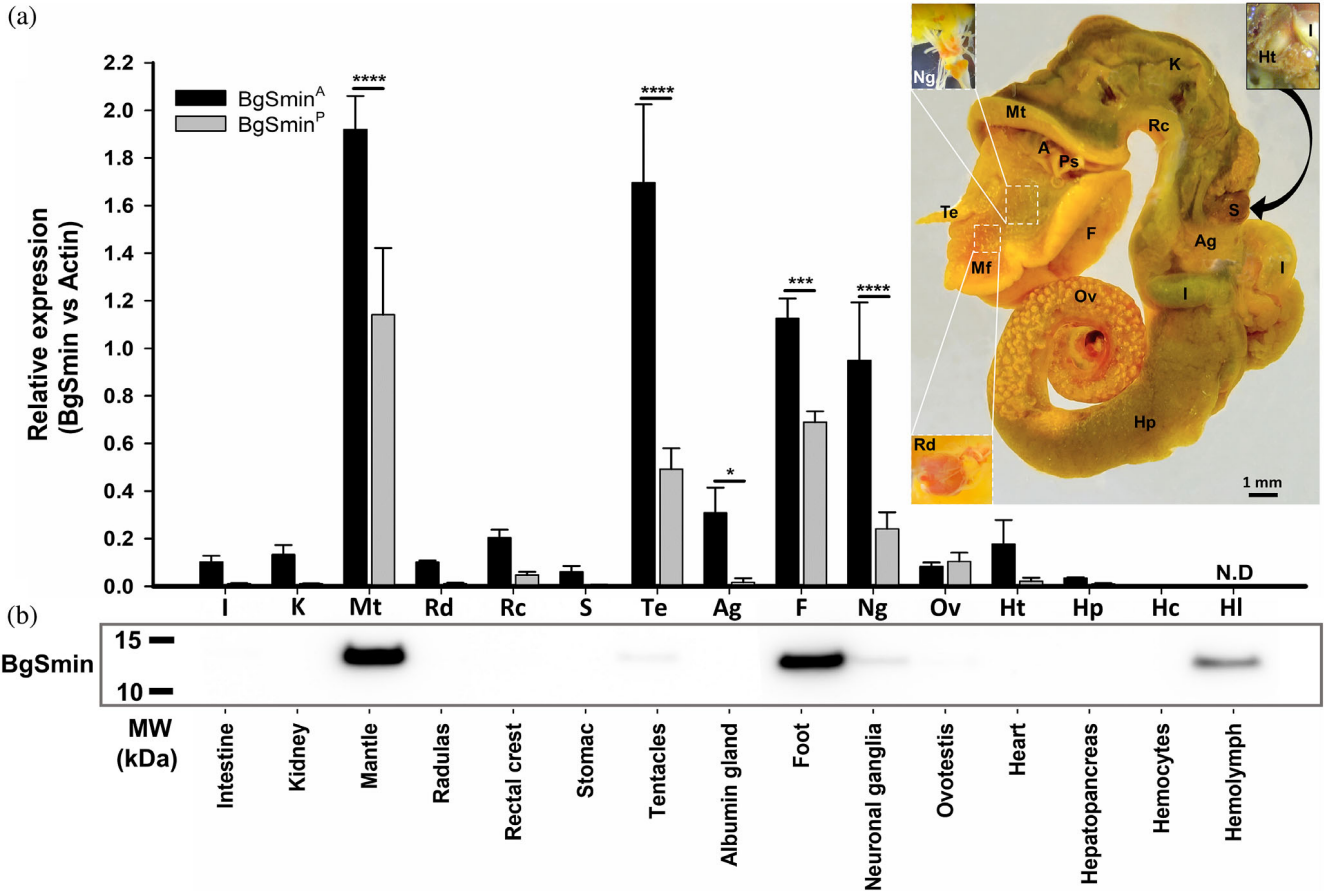
Tissue distribution of schistosomin transcripts and protein in *B. glabrata*. (a) Isoform‐specific RT‐qPCR analysis of schistosomin expression across *B. glabrata* tissues. Data correspond to three independent biological preparations, each analyzed in technical duplicate. Relative transcript levels of BgSmin^A^ (black bars) and BgSmin^P^ (gray bars) were quantified and normalized to actin expression. Data are presented as mean ± SD. Statistical comparisons between BgSmin^A^ and BgSmin^P^ within each tissue were performed using two‐way ANOVA followed by Šídák's multiple comparisons test in GraphPad Prism (v.10); statistical significance was defined as follows: *p < .05; **p < .01; ***p < .001; **** < 0.0001 (*). The anatomical origin of each sampled tissue is indicated on the dissected *B. glabrata* specimen and enlarged insets. Ag, albumen gland; A, anus; F, foot; Ht, heart; Hc, hemocytes; HI, hemolymph; Hp, hepatopancreas; I, intestine; K, kidney; Mt, mantle; Mf, mantle fold; Ng, neuronal ganglia; Ov, ovotestis; Ps, pseudobranch; Rd, radula; Rc, rectal crest; S, stomach; Te, tentacles. Anus, mantle fold, and pseudobranch were not included in the analyses shown in panels (a and b). Hemocytes were collected from the hemolymph of 80 snails. No RNA was extracted from hemolymph and RT‐qPCR was therefore not performed (N.D., not determined). (b) Western blot analysis of schistosomin in selected tissues. Protein extracts were obtained from the same biological samples used for the RT‐qPCR analysis. Hemolymph samples were analyzed in parallel. A single immunoreactive band with an apparent molecular mass between 10 and 15 kDa was detected, consistent with the expected size of the mature schistosomin protein.

We examined the tissue distribution of schistosomin at both the transcript and protein levels, taking advantage of the isoform‐specific qPCR strategy and the antibodies generated using the synthetic protein. Quantitative isoform‐specific RT‐qPCR analyses allowed discrimination between the two schistosomin isoforms, BgSmin^A^ and BgSmin^P^, and revealed a marked tissue‐specific expression pattern. The highest transcript levels were detected in the foot, nervous ganglia, mantle, and tentacles, whereas hepatopancreas and hemocytes exhibited low or undetectable expression levels (Figure 6a). Importantly, in all tissues analyzed, the BgSmin^A^ isoform was consistently and significantly more highly expressed than isoform P. However, no tissue displayed exclusive expression of either isoform, indicating that both variants are co‐expressed across tissues, albeit at different relative levels. Overall, this transcriptional profile indicates that schistosomin expression is not restricted to neuronal tissues but extends to several peripheral and epithelial compartments.

At the protein level, Western blot analyses detected schistosomin in the foot and nervous ganglia, but also in the mantle and tentacles (Figure 6b). While no signal was observed in hepatopancreas or hemocytes, schistosomin was readily detected in the hemolymph. This apparent discrepancy between low transcript levels and detectable protein abundance in the hemolymph is consistent with the secreted nature of schistosomin. Indeed, the protein contains a signal peptide and has previously been reported as a circulating factor in snail hemolymph (Hordijk, van Loenhout, et al., 1991; Joosse et al., 1988). These observations suggest that schistosomin is synthesized in specific tissues and subsequently released into the circulatory system, where it can distribute independently of local transcriptional activity, thereby partially decoupling mRNA abundance from protein levels measured by immunoblotting. Notably, the protein‐level data reflect the overall tissue distribution of schistosomin rather than isoform‐specific expression patterns because Western blot analysis cannot distinguish the two schistosomin isoforms.

## 3 DISCUSSION

Here, we successfully implemented a total chemical synthesis strategy to obtain sufficient quantities of homogeneous material of schistosomin (Smin) from *Biomphalaria glabrata* to overcome either the limited amount of material obtainable from snail tissues or the limitations of recombinant expression systems, which previously yielded insoluble or heterogeneous material. We introduced a C‐terminal biotinylated lysine for functional studies and used an optimized oxidative folding protocol, involving glutathione‐based redox buffers and low‐temperature conditions, to ensure the production of homogeneous, correctly folded protein suitable for structural and biophysical analyses. This synthetic approach not only facilitated the current study but also provides a template for producing other cysteine‐rich miniproteins that are challenging to express recombinantly.

Through a combination of total chemical synthesis and high‐resolution X‐ray crystallography, we have elucidated the three‐dimensional architecture of BgSmin as a novel and robust scaffold with unique properties. Indeed, the crystal structures of BgSmin^Synth^ reveal a previously undescribed disulfide‐rich fold, characterized by four intramolecular disulfide bridges (Cys22‐Cys62, Cys31‐Cys55, Cys41‐Cys76, and Cys52‐Cys85) that define a 1–6, 2–5, 3–7, and 4–8 connectivity pattern. Such a topology is expected to contribute to the intrinsic stability of the scaffold, in agreement with previous reports describing the resistance of native schistosomin to thermal treatment in *L. stagnalis* (Hordijk, van Loenhout, et al., 1991). Here, by nanoDSF and CD, we validated the exceptional stability of Smin, with a high temperature (Ti_2_), which is quite unexpected in the natural biotopes of these tropical snails. The marked destabilization observed under reducing conditions indicates, as expected, that this thermal stability strongly depends on the conserved disulfide‐bond network. Therefore, this topology, so far unknown in the Protein Data Bank, confers remarkable rigidity to the scaffold.

Despite low overall sequence identity, the structural conservation observed across schistosomin‐like sequences including cone snail peptides supports the hypothesis that the disulfide framework is the primary determinant of the fold stability. AF3 predictions for several schistosomin‐like peptides from diverse gastropod species confirmed a shared compact architecture, reinforcing the notion that this scaffold represents a broadly distributed family of disulfide‐rich miniproteins. These results highlight a remarkable combination of structural conservation and diversification: schistosomin‐like proteins have diverged extensively at the sequence level while preserving a highly conserved disulfide‐stabilized fold. The presence of distinct structural clusters within this conserved framework suggests that evolutionary diversification has occurred under strong structural constraints, likely imposed by the stability and functional requirements of the scaffold. These observations establish schistosomin as the prototype of a broadly distributed family of disulfide‐rich miniproteins whose architecture is encoded by the cysteine disulfides. They further provide a strong rationale for expanding the repertoire of Smin‐like peptides and integrating structural, phylogenetic, and functional approaches to investigate their evolutionary trajectories and biological roles across mollusks.

On the basis of sequence features and cysteine topology, several peptides of cone snail are currently classified in ConoServer as class XXII conotoxins (Li et al., 2025). However, this assignment remains purely descriptive, as members of this class are largely uncharacterized and lack functional annotation. Importantly, the presence of closely related sequences in non‐venomous gastropods strongly suggests that these peptides should not be viewed as canonical conotoxins, but rather as representatives of a broader and previously unrecognized family of disulfide‐rich molluskan miniproteins.

Although schistosomin was originally described as a neuroendocrine factor potentially involved in host–parasite interactions and parasitic castration in *L. stagnalis* (Hordijk, van Loenhout, et al., 1991; Schallig et al., 1991), its precise biological function in *B. glabrata* remains unresolved (Zhang et al., 2009). Moreover, we showed that two BgSmin isoforms differing by a single Ala/Pro substitution at position 78 co‐exist in the mollusk. Quantitative RT‐qPCR and Western blot analyses revealed that schistosomin transcripts and proteins are not restricted to neuronal tissues but are also abundant in peripheral and epithelial compartments, such as the foot, mantle, and tentacles. These results demonstrate that schistosomin is expressed in multiple tissues and circulates within *B. glabrata*. Remarkably, strong expression in the foot, mantle, and tentacles, tissues that are directly exposed to the external environment, raises the possibility that schistosomin may participate in interactions with surrounding biological agents such as microorganisms or parasites. The absence of a strictly neuron‐specific expression pattern, combined with its secreted and systemic distribution, argues against a classical neuropeptide function (Li et al., 2026; Okyem et al., 2025; Ramirez et al., 2024) and indicates that the physiological role of schistosomin in this gastropod species remains to be elucidated.

In this respect, *B. glabrata* provides access to schistosomin‐like peptides expressed in non‐venomous tissues and physiological contexts, in contrast to cone snails, where related disulfide‐rich peptides are predominantly studied in the framework of venom specialization. Moreover, the availability of well‐established laboratory breeding conditions enables reproducible access to biological material, making this organism a tractable experimental model to investigate the structural, evolutionary, and functional properties of this family independently of venom‐associated constraints. The tools developed in this study therefore provide a robust foundation to address the long‐standing question of schistosomin‐like protein function in gastropods and in the host–parasite relationship.

## 4 CONCLUSION

In summary, the structural relationship between schistosomin and peptides currently classified as class XXII conotoxins suggests that this scaffold may be more widespread in mollusks than previously recognized. Future studies integrating structural, phylogenetic, and functional analyses will be essential to elucidate the evolutionary origins and biological roles of schistosomin‐like proteins across gastropod species. Finally, this work establishes schistosomin as a novel miniprotein scaffold with exceptional stability and provides a comprehensive structural and biological framework for further exploration of its potential in biotechnology and medicine. During revision of this manuscript, Hackney et al. reported the structural and functional characterization of Conkazal‐M1, a MKAVA‐family conotoxin from *Conus magus* adopting a Kazal‐type protease inhibitor fold, and noted its structural similarity to schistosomin (Hackney et al., 2026). This independent observation further supports the view that schistosomin belongs to a broader group of structurally related disulfide‐rich molluskan miniprotein scaffolds.

## 5 MATERIALS AND METHODS

### 5.1 Snail maintenance

*Biomphalaria glabrata* snails (Puerto Rico strain) were maintained under controlled laboratory conditions in aerated aquaria containing deionized water at 28°C. Animals were fed ad libitum with fresh spinach and a mixture of spirulina, brewer's yeast, skimmed milk, and wheat germ under a 12 h light/12 h dark cycle and starved for 24 h prior to tissue collection.

### 5.2 Native schistosomin extraction and enrichment

For native schistosomin purification, 30 adult snails (17–20 mm shell diameter) were anesthetized in chilled 50 mM MgCl_2_, and foot tissues, which contain the neuronal ganglia, were dissected, immediately frozen in liquid nitrogen, and ground to a fine powder using a pre‐chilled mortar and pestle. Frozen foot tissues were homogenized in 12 mL of ultrapure water and sonicated (30 cycles of 30 s on/off) at 4°C using a Bioruptor® Plus (Diagenode) equipped with an integrated cooling system. The homogenate was centrifuged at 20,000 × g for 20 min at 4°C, and the supernatant was collected and split into two 6 mL fractions. Proteins were sequentially precipitated by ammonium sulfate fractionation. Samples were first brought to 60% ammonium sulfate saturation and incubated for 30 min at room temperature, followed by centrifugation at 15,000 × g for 30 min at 4°C. The resulting supernatant was then brought to 80% ammonium sulfate saturation, and the precipitated proteins were collected by centrifugation. Final protein pellets were resuspended in 500 μL of 10 mM HEPES buffer (pH 7.5) and dialyzed at 4°C against 500 mL of the same buffer using 3.5 kDa molecular weight cut‐off Slide‐A‐Lyzer™ cassettes (Thermo Fisher Scientific), with three buffer changes over 6 h. Samples were stored at −20°C until further use. Protein concentrations were determined by BCA assay (Thermo Fisher Scientific, #A55865) prior to chromatographic analysis.

### 5.3 Purification of native BgSmin^A^ and BgSmin^P^

Dialyzed protein extracts were subjected to reversed‐phase HPLC purification on a Gilson PLC‐2020 system equipped with an XBridge Peptide BEH C18 OBD Prep column (300 Å, 5 μm, 10 × 250 mm). Proteins were eluted at 50°C using a linear gradient of acetonitrile in water containing 0.1% (v/v) trifluoroacetic acid (0%–10% B for 5 min, then 15%–40% B over 60 min) at a flow rate of 5 mL·min^−1^, with UV detection at 215 nm. Fractions corresponding to the two major schistosomin peaks were collected separately and used for subsequent UPLC–MS and HRMS analyses. For analytical characterization of purified fractions, 2 μL of protein solution at 1 mg·mL^−1^ was injected.

### 5.4 UPLC–MS and HRMS analysis

Protein extracts and purified schistosomin fractions were analyzed by UPLC–MS using a Thermo Scientific Dionex Ultimate 3000 system coupled to an LCQ Fleet mass spectrometer. Separations were performed on a reversed‐phase C18 column (ACQUITY UPLC Peptide BEH, 300 Å, 1.7 μm, 2.1 × 150 mm) using a linear gradient of 0%–70% acetonitrile in water (both containing 0.1% (v/v) trifluoroacetic acid) over 15 min, at a flow rate of 0.4 mL·min^−1^ and a column temperature of 70°C. Detection was carried out by UV absorbance at 215 nm and mass spectrometry in positive ion mode. Source settings were: ion transfer temperature 350°C, spray voltage 2.8 kV, capillary temperature 350°C, capillary voltage 10 V, and tube lens voltage 75 V.

High‐resolution mass spectrometry (HRMS) analyses of purified schistosomin isoforms were performed using an Orbitrap ID‐X mass spectrometer (Thermo Scientific) coupled to a Vanquish UPLC system, equipped with a Kinetex EVO C18 column (50 × 2.1 mm, 1.7 μm, Phenomenex). UV–Vis chromatograms were recorded between 200 and 400 nm. Mass spectra were acquired in the 100–2000 m/z range at a resolution of 120,000 using an electrospray ionization (ESI) source operating in positive mode.

### 5.5 Chemical synthesis of schistosomin

Schistosomin was produced by total chemical synthesis using a strategy combining solid‐phase peptide synthesis (SPPS), chemoselective ligation, and controlled oxidative folding. The full‐length protein (residues 18–96) was assembled from three peptide segments corresponding to segment A (DNYRCPNPGDAFECFESDATARF), segment B (CVSGKRGAYVICSKCRRKYEF), and segment C (CANGAKVSKRPEVECRADWASTECTSENSDVPSVM‐K(Biot)‐NH_2_), all prepared by Fmoc‐based SPPS, with peptide thioesters being synthesized using a combination of Fmoc‐based SPPS and solution‐phase chemistry (Ollivier et al., 2010). For chemical assembly, segment A was converted into a peptide thioester, whereas segment B was prepared as a SEA^off^ peptide and segment C as a C‐terminal peptide amide. To prevent aspartimide formation during synthesis, Asp88 was substituted by Glu. In addition, a C‐terminal lysine bearing a biotin moiety was introduced to facilitate downstream functional studies.

#### 5.5.1 Assembly of schistosomin

##### NCL (step 1)

Peptide thioester **1** (13.0 mg, 4.2 μmoles) and SEA^off^ peptide **2** (15.5 mg, 4.2 μmoles) were dissolved in 0.1 M sodium phosphate buffer, pH 7.2, containing 6 M Gn.HCl, 200 mM 4‐mercaptophenylacetic acid (MPAA), and 10 mM n‐octyl glucoside. The reaction mixture was agitated at 37°C overnight.

An aliquot (5 μL) was diluted with 20% aqueous acetic acid (95 μL) and extracted with diethyl ether three times to remove the excess of MPAA before UPLC–MS analysis. Then, TCEP·HCl (5 μL, 200 mM in water, final concentration 10 mM) was added to this aliquot, which was kept at room temperature for 1 h. UPLC–MS analysis of this sample showed the successful formation of intermediate **3**, which eluted in its reduced form **4**.

##### SEA‐mediated ligation (step 2)

The SEA group of intermediate **3** was reduced and activated by adding TCEP·HCl to the reaction mixture at a final concentration of 80 mM and adjusting the pH to 5.5 using 6 M HCl. Peptide segment 5 (21.8 mg, 4.65 μmoles), solubilized in 0.1 M sodium phosphate buffer, pH 5.5, containing 6 M Gn.HCl, 200 mM MPAA, and 10 mM n‐octyl glucoside (840 μL), was then added. The reaction mixture was agitated at 37°C for 44 h.

Aliquots (10 μL) were diluted with 20% aqueous acetic acid (95 μL) and extracted with diethyl ether three times to remove the excess of MPAA before UPLC–MS analysis.

#### 5.5.2 HPLC purification

The reaction mixture was diluted with aqueous acetic acid (10 mL, 20% in deionized water) and then extracted with diethyl ether to remove the excess of MPAA (2 mL × 16). The excess of diethyl ether was removed by bubbling argon into the solution for 15 min. The crude peptide was purified using an XBridge BEH C18 column (300 Å, 5 μm, 10 × 250 mm), at a flow rate of 6 mL·min^−1^ and a temperature of 65°C. Eluent A was 0.10% TFA in water and eluent B was 0.10% TFA in CH_3_CN. The gradient was 0%–10% B over 5 min, followed by 10%–40% B over 60 min.

The purified fractions were collected, frozen, and lyophilized to provide 19.98 mg (45.2%) of schistosomin linear precursor.

#### 5.5.3 Folding of schistosomin

The folding of schistosomin linear precursor was performed at 4°C. Schistosomin linear precursor (11 mg) was dissolved in PBS buffer containing 6 M Gn.HCl (1.1 mL). This solution was diluted with PBS buffer containing glycerol as a co‐solvent (10% by volume), n‐octylglucoside (2 mM), and the redox system GSH (1 mM)/GSSG (0.2 mM). The folding mixture was gently agitated for 32 days.

The folded schistosomin product was purified by HPLC as described above for the linear precursor. The collected fractions were pooled, frozen, and lyophilized to provide 2.3 mg (23%) of folded schistosomin.

An independent synthesis and folding preparation yielded 0.85 mg of folded BgSmin^Synth^ with a final isolated yield of 17.4%, supporting the reproducibility of the procedure (Figure S6). The folding process yielded a predominant species corresponding to the correctly folded schistosomin, as confirmed by UPLC‐MS and high‐resolution mass spectrometry (Figures S4 and S5). Experimental procedures and analytical characterization are provided in the Supplementary Information (Figures S2–S5).

### 5.6 Crystallization and structure determination

Crystallization conditions for the synthetic schistosomin were initially screened at a protein concentration of 10 mg·mL^−1^ using commercial kits (Qiagen) and a Mosquito nanoliter dispensing system (SPT Labtech, Melbourn, UK). Crystals were manually reproduced in hanging‐drop vapor diffusion experiments by mixing equal volumes of protein and reservoir solutions. The same crystallization condition yielded two distinct crystal forms. Crystals were cryoprotected by transfer into mother liquor supplemented with 25% sucrose and flash‐cooled in liquid nitrogen.

Diffraction data were collected at 100 K on the PROXIMA 1 and PROXIMA 2 beamlines at Synchrotron SOLEIL (Saint‐Aubin, France). Diffraction intensities were integrated by the XDS program (Kabsch, 2010) using the autoPROC pipeline (www.globalphasing.com), including STARANISO anisotropy correction (Vonrhein et al., 2011). Structures were solved by molecular replacement with PHASER (McCoy et al., 2007) using an AlphaFold2 model as a search model. Refinement of each structure was performed with BUSTER (Blanc et al., 2004), using TLS group and NCS restraints when necessary. Inspection of the density maps and manual rebuilding were performed using COOT (Emsley & Cowtan, 2004). Refinement details of each structure are shown in Table 1. Molecular graphic images were generated using PyMOL (http://www.pymol.org).

### 5.7 Biophysical characterization

The concentration of synthetic schistosomin solubilized in 10 mM sodium phosphate buffer (pH 7.2) was determined by UV absorbance at 280 nm using a NanoDrop spectrophotometer, based on its theoretical extinction coefficient (*ε* = 10,470 L·mol^−1^·cm^−1^). For circular dichroism (CD) measurements, the stock solution was diluted to 0.1 mg·mL^−1^ in the same buffer.

CD spectra were recorded on a Jasco J‐815 spectropolarimeter equipped with a PFD‐425S Peltier temperature control unit, using a 0.1 cm path‐length quartz cuvette (Hellma). Spectra were acquired between 185 and 260 nm at 25°C with a bandwidth of 2 nm, a data pitch of 1 nm, and an integration time of 1 s. Each spectrum corresponded to the average of eight accumulations and was smoothed using a five‐point algorithm. CD data were expressed as mean residue weight ellipticity. Thermal denaturation experiments were performed using the same settings, with six accumulations per point, by increasing the temperature from 15 to 90°C in 5°C increments, with a 1 min equilibration time at each step. Ellipticity values at 222 nm were monitored as a function of temperature.

Nano‐differential scanning fluorimetry (nanoDSF) experiments were carried out on a Prometheus NT.48 instrument (NanoTemper Technologies) using 5 μL of BgSmin^Synth^ at 30 μM in 10 mM sodium phosphate buffer (pH 7.4). For reducing conditions, BgSmin^Synth^ was supplemented with 1 mM DTT and incubated for 20 min on ice before loading into nanoDSF capillaries. Samples were heated from 20 to 90°C at a rate of 2.5°C·min^−1^. Three technical replicates were analyzed in the absence of DTT and four technical replicates in the presence of DTT, using aliquots from the same protein preparation. The intrinsic fluorescence ratio (350/330 nm) was monitored as a function of temperature.

Thermal denaturation data obtained from CD and nanoDSF were analyzed using sigmoidal regression models implemented in SigmaPlot (v14.5). NanoDSF and CD data were analyzed without smoothing. A single‐transition model was described by the equation *f*(*T*) = *A* + *A*_1_/(1 + exp(−(*T* – Ti_1_)/*k*_1_)), where *A* corresponds to the baseline signal, *A*_1_ is the amplitude of the transition, Ti_1_ is the apparent inflection temperature, and *k*_1_ is a slope factor related to transition cooperativity. Biphasic unfolding behavior was fitted using the double‐sigmoidal equation *f*(*T*) = *A* + *A*_1_/(1 + exp(−(*T* − Ti_1_)/*k*_1_)) + *A*_2_/(1 + exp(−(*T* − Ti_2_)/*k*_2_)), where Ti_1_ and Ti_2_ correspond to the apparent inflection temperatures of the two transitions, and *A*_1_, *A*_2_, *k*_1_, and *k*_2_ define their respective amplitudes and slope parameters. Model quality was assessed by residual analysis.

### 5.8 Molecular dynamics simulations

Molecular dynamics (MD) simulations were performed using the GROMACS 2023.4 package (Van Der Spoel et al., 2005) and the CHARMM36 force field (Huang & MacKerell Jr., 2013) with the TIP3P water model. The starting structure of BgSmin^A^ D88E was derived from the X‐ray crystal structure of the synthetic protein (PDB 9FDO, chain B), after removal of the C‐terminal Lys(Biot) residue using ChimeraX. The BgSmin^P^ D88E variant was generated in silico by substituting Ala78 for Pro78.

The protein was placed in a dodecahedral simulation box with a minimum distance of 2.0 nm between the solute and the box boundaries. The system was solvated with explicit water molecules, neutralized, and adjusted to an ionic strength of 70 mM NaCl. The N‐terminus was protonated and the C‐terminus treated as deprotonated. Energy minimization was performed until the maximum force was below 1000 kJ·mol^−1^·nm^−1^, followed by 400 ps equilibration under position restraints at 1 bar and 300 K. Production runs were performed under NPT conditions using a velocity‐rescale thermostat and a Parrinello–Rahman barostat. Long‐range electrostatic interactions were treated using the particle mesh Ewald method with a Fourier grid spacing of 0.12 nm, interpolation order 4, and a relative tolerance of 10^−5^. Van der Waals interactions were treated using a force‐switch cutoff scheme with a switching distance of 1.0 nm and a cutoff distance of 1.2 nm. Each simulation was performed in triplicate with a time step of 2 fs, coordinates saved every 10 ps, and a total simulation time of 160 ns per trajectory.

Structural stability and dynamics were assessed using root‐mean‐square fluctuation (RMSF).

### 5.9 Multiple alignments and structure superimpositions

Schistosomin and schistosomin‐like sequences were retrieved from public databases. The dataset included the following sequences: *Biomphalaria glabrata* Smin^A^ (NP_001298209.1), *B. glabrata* Smin^P^ (XP_055889456.1), *Bulinus truncatus* (KAH9500249), *Lymnaea stagnalis* (CAL1528904), *Aplysia californica* Smin1 (NP_001191584) and Smin2 (XP_005098364), *Elysia marginata* (GFS27466), *Elysia chlorotica* (RUS82021), *Physella acuta* Smin1 (XP_059170191) and Smin2 (XP_059170187), *Plakobranchus ocellatus* (GFO13819). *Conus ebraeus* (DAZ86321), *Conus geographus* (BAO65647). For the other cone species, the sequences are only listed and named on the ConoServer: *Conus litteratus* (Lt22.2), *Conus tulipa* (T22.1), *Conus bayani* (Ba22.1) and *Conus cactus* (C22.1).

Predicted signal peptides were removed, and multiple sequence alignment of the mature peptide sequences was performed using Clustal Omega through the UniProt alignment tool (Sievers et al., 2011; Zaru et al., 2023).

Structural models were generated from the corresponding amino acid sequences using AlphaFold3. Structural similarity between predicted models was assessed using the DALI server, which calculates pairwise *Z*‐scores reflecting structural similarity between protein structures (Holm et al., 2023). In addition, structural alignments and superimpositions were performed using Foldseek to visualize conserved structural features across the dataset (van Kempen et al., 2024).

### 5.10 Gene expression and protein localization

Adult *Biomphalaria glabrata* snails were anesthetized by incubation for 10–20 min in chilled 50 mM MgCl_2_. Soft tissues were removed from the shell, rinsed in snail phosphate‐buffered saline (8.41 mM Na_2_HPO_4_, 1.65 mM NaH_2_PO_4_·H_2_O, 45.34 mM NaCl, pH 7.2), and dissected under a Zeiss stereomicroscope in a Sylgard‐coated Petri dish. The following tissues were collected: tentacles, mantle collar, hepatopancreas, gonads, intestine, kidneys, rectal crest, foot, heart, stomach, radula, and neuronal ganglia. Tissues were pooled in groups of three and transferred into Lysing Matrix D tubes (MP Biomedicals) containing 1 mL of TRIzol reagent (Thermo Fisher Scientific, #15596026). All dissections were performed in triplicate.

Hemolymph was collected from 80 snails (15–20 mm shell diameter) by foot puncture using 200 μL gel‐loading tips. Hemolymph from 10 individuals was pooled per sample and transferred into 12‐well culture plates, then incubated at 26°C for 3 h to allow hemocyte adhesion. Hemocyte‐free supernatants were collected and mixed with TRIzol LS reagent (Thermo Fisher Scientific, #10296028), whereas adherent hemocytes were washed three times with snail phosphate‐buffered saline and processed using the NucleoSpin RNA/Protein kit (Macherey‐Nagel, #740933.50).

Total RNA was reverse‐transcribed using the AffinityScript Multi Temperature cDNA Synthesis kit (Agilent, #200436) according to the manufacturer's instructions, using 500 ng of total RNA per reaction. Quantitative PCR (qPCR) was performed using the Brilliant III Ultra‐Fast SYBR Green QPCR Master Mix (Agilent, #600882) in a final volume of 20 μL in 96‐well optical plates (Axygen, PCR‐96‐FLT‐C). Each reaction contained 250 nM of each primer (Table S2) and 1 μL of cDNA. Amplification was carried out on a QuantStudio 3 system (Applied Biosystems) using the following cycling conditions: 95°C for 5 min, followed by 40 cycles of 95°C for 10 s and 60°C for 20 s, followed by a melting‐curve analysis. Relative expression levels of schistosomin isoforms were normalized to actin expression and calculated using the 2^‒ΔΔCt^ method. Statistical comparisons within each tissue were performed using two‐way ANOVA followed by Šídák's multiple comparisons test in GraphPad Prism (v.10). Experiments were performed in three independent biological preparations, each analyzed in technical duplicate.

For antibody production, a synthetic schistosomin linear precursor was used to immunize three female NMRI mice by subcutaneous injection of 75 μg peptide emulsified in alum adjuvant (1:1, v/v; 200 μL total volume) on days 0, 21, and 31. Terminal serum was collected 35 days after the last injection and schistosomin detection assessed by Western blot against native and synthetic proteins.

For Western blot analysis, protein concentrations were determined using the BCA Protein Assay Kit (Thermo Fisher Scientific, #A55865). Equal amounts of protein (20 μg per sample) were separated under reducing conditions on NuPAGE 4%–12% Bis‐Tris gels (Invitrogen, #NP0322BOX) and transferred for 1.5 h at 65 V onto 0.45 μm PVDF membranes (Immobilon‐P®, Millipore, #IPVH00010) in Towbin buffer containing 10% methanol and 0.0025% SDS. BgSmin^Synth^ (5, 10, or 30 ng) was included as control. Membranes were blocked for 1 h at room temperature in blocking buffer (8 g·L^−1^ high‐purity casein, PBS, 0.2% Tween‐20), incubated overnight at 4°C with anti‐schistosomin primary antibody (1:2000 in PBS containing 5% protease‐free BSA and 0.1% sodium azide), washed three times in PBS/0.05% Tween‐20, and incubated for 1 h at room temperature with HRP‐conjugated anti‐mouse secondary antibody (Jackson ImmunoResearch, #115‐035‐146) diluted 1:50,000. Detection was performed using SuperSignal West Dura substrate (Thermo Scientific, #37075), and signals were recorded on an ImageQuant system (Cytiva).

## AUTHOR CONTRIBUTIONS

**Oleg Melnyk:** Conceptualization; investigation; methodology; data curation; supervision; visualization; funding acquisition; writing – original draft; writing – review and editing; project administration; validation; formal analysis. **Benoît Snella:** Investigation; methodology; data curation. **Aurélie Parmentier:** Methodology; resources. **Alexandra Mougel:** Investigation; methodology; visualization. **Céline Boidin‐Wichlacz:** Conceptualization; methodology; visualization; supervision. **Stéphanie Caby:** Methodology; investigation; writing – review and editing; visualization; resources. **Jérôme Vicogne:** Conceptualization; methodology; data curation; supervision; resources; project administration; investigation; validation; visualization; funding acquisition; writing – original draft; writing – review and editing; formal analysis. **Christine Demanche:** Methodology; investigation; writing – review and editing; data curation; formal analysis; visualization; resources. **Magalie Sénéchal:** Methodology; data curation; visualization. **Rémi Desmet:** Methodology; data curation; visualization. **Ugo Pasco:** Investigation; methodology; data curation. **Armelle Vigouroux:** Methodology; visualization; writing – review and editing; data curation. **Solange Moréra:** Conceptualization; investigation; methodology; data curation; supervision; formal analysis; validation; visualization; funding acquisition; writing – original draft; writing – review and editing; project administration. **Sonia Cantel:** Methodology; visualization; writing – original draft; investigation.

## CONFLICT OF INTEREST STATEMENT

The authors declare no conflicts of interest.

## Supporting information


**Appendix S1:** Supporting information.

**Figure S1.** Purification, UPLC‐MS and HRMS of BgSmin^A^ and BgSmin^P^ from *B. glabrata*.
**Figure S2:** General strategy for accessing schistosomin by chemical synthesis.
**Figure S3:** UPLC–MS analysis of purified schistosomin linear precursor.
**Figure S4:** UPLC–MS analysis of folded schistosomin after HPLC purification.
**Figure S5:** LC–HRMS analysis of synthetic schistosomin.
**Figure S6:** Repeatability of the oxidative folding of synthetic schistosomin.
**Figure S7:** Thermal denaturation profile of BgSmin^Synth^ monitored by circular dichroism.
**Figure S8:** Molecular dynamics simulation.
**Figure S9:** Schistosomin and conotoxin predicted structures.
**Table S1:** Pairwise structural similarity matrix based on *Z*‐scores from the DALI server.
**Table S2:** Primer sequences for qPCR analysis.

## Data Availability

The data supporting the findings of this study are available within the article, its Supplementary Information, and the associated Data Source file. Structural data have been deposited in the Protein Data Bank (PDB) under accession codes 9RT6 and 9FDO. Additional datasets generated and/or analyzed during the current study are available from the corresponding authors upon reasonable request.
