# Supplementary material for: Total synthesis and structural characterization of a novel protein scaffold from the snail Biomphalaria glabrata
Source: Protein Sci. 2026 Aug 5;35(9):e70745. doi: 10.1002/pro.70745 (PMC13440164; doi:10.1002/pro.70745)
Supplement: Supplementary file 1 [file PRO-35-e70745-s001.docx]

**Supplementary file for:**

**Total synthesis and structural characterization of a novel protein scaffold
 from the snail *Biomphalaria glabrata***

Oleg Melnyk^1^, Stéphanie Caby§,^1^ Armelle Vigouroux§,^2^ Christine Demanche, ^1^ Rémi Desmet,^1^ Magalie Sénéchal,^1^ Benoît Snella,^1^ Alexandra Mougel,^1^ Céline Boidin-Wichlacz,^1^ Aurélie Parmentier,^1^ Ugo Pasco,^3^ Sonia Cantel,^3^ Solange Moréra*^2^, Jérôme Vicogne*^1^

^1^ Univ. Lille, CNRS, INSERM, CHU Lille, Institut Pasteur de Lille, U1019 - UMR 9017 - CIIL - Center for Infection and Immunity of Lille, F-59000 Lille, France.

^2^ Université Paris-Saclay, CEA, CNRS, Institute for Integrative Biology of the Cell (I2BC), 91198, Gif-sur-Yvette, France

^3^ IBMM, Univ. Montpellier, ENSCM, CNRS, Montpellier, France.

§ Contributed Equally to this work

* Correspondence:

Solange Moréra: CEA, CNRS, Université Paris-Saclay, Institute for Integrative Biology of the Cell (I2BC), F-91198 Gif-sur-Yvette, France E-mail: [solange.morera@i2bc.paris-saclay.fr](mailto:solange.morera@i2bc.paris-saclay.fr)

Jérôme Vicogne : Univ. Lille, CNRS, INSERM, CHU Lille, Institut Pasteur de Lille, U1019 – UMR9017, CIIL - Centre for Infection and Immunity of Lille d’infection et l’immunité de Lille, F-59000, Lille, France. E-mail: [jerome.vicogne@cnrs.fr](mailto:jerome.vicogne@cnrs.fr)

**Figure S1.** **Purification, UPLC-MS and HRMS of BgSmin^A^ and BgSmin^P^ from *B. glabrata.***

**A.** UPLC-MS analysis of Bg protein extracts was performed using Thermo Scientific Dionex ultimate 3000 HPLC (top) coupled to LCQ Fleet MS detector (bottom; Column: ACQUITY UPLC Peptide BEH C18, 300 Å, 1.7 µm, 2.1 mm x 150 mm, gradient 0 - 70 % B in 15 min at 70 °C, 0.4 mL/min. Eluent A: Water + 0.1 % v/v TFA. Eluent B: Acetonitrile + 0.1 % v/v TFA.

**B. UPLC analysis of purified BgSmin^A^ (top) and BgSmin^P^ (bottom).**

Separation of proteins has been performed using an HPLC system: Gilson PLC-2020 equipped with column xBridge Peptide BEH C18 OBD Prep Column, 300 Å, 5 µm, 10 mm × 250 mm. Gradient: 0-10% B in 5 min, then 15-40% B in 60 min. Eluent A: Water + 0.1 % v/v TFA. Eluent B: Acetonitrile + 0.1 % v/v TFA, 5 mL/min, temperature: 50 °C, detection at 215 nm, 2 µL of a protein solution at 1 mg/mL was injected for the analysis)

BgSmin^P^

BgSmin^A^

**C. HRMS analysis of purified BgSmin^A^.**

HRMS data were acquired using an Orbitrap ID-X (Thermo) with ESI source coupled with an UPLC Vanquish (Thermo), with a Kinetex EVO C18 50 mm x 2.1 mm 1.7 µm column (Phenomenex). UV-Vis chromatograms were acquired on a 200-400 nm range. Mass spectra were acquired in a 100-2000 Da range in positive mode with a 120.000 resolution. ESI, *m/z* for [C_369_H_570_N_110_O_122_ S_10_]^8+^ calculated m/z for A+1 ion: 1097.8656, measured: 1097.8656.

**D. HRMS analysis of purified BgSmin^P^.**

HRMS data were acquired using an Orbitrap ID-X (Thermo) with ESI source coupled with an UPLC Vanquish (Thermo), with a Kinetex EVO C18 50 mm x 2.1 mm 1.7 µm column (Phenomenex). UV-Vis chromatograms were acquired on a 200-400 nm range. Mass spectra were acquired in a 100-2000 Da range in positive mode with a 120.000 resolution. ESI, m/z for [C_371_H_564_N_110_O_122_S_10_]8+ calculated m/z for A+2 ion: 1101.2427, measured: 1101.2429.

**Figure S2. General strategy for accessing schistosomin by chemical synthesis.**

**Figure S3. UPLC-MS analysis of purified schistosomin linear precursor.**

A. LC trace, eluent A 0.10% TFA in water, eluent B 0.10% TFA in CH_3_CN. Acquity BEH C18 column, 300 Å, 1.7 μm (2.1 × 100 mm) column, gradient 0-70% B in 20 min, 70 °C, 0.4 mL/min, detection at 215 nm. B. MS trace.

**A**

**B**

**Figure S4. UPLC-MS analysis of folded schistosomin after HPLC purification.**

A. LC trace, eluent A 0.10% TFA in water, eluent B 0.10% TFA in CH_3_CN. Acquity BEH C18 column, 300 Å, 1.7 μm (2.1 × 100 mm) column, gradient 0-70% B in 20 min, 70 °C, 0.4 mL/min, detection at 215 nm. B. MS trace.

**A**

**B**

**Figure S5. LC-HRMS analysis of synthetic schistosomin**.

HRMS data were acquired using an Orbitrap ID-X (Thermo) with ESI source coupled with an UPLC Vanquish (Thermo), with a Kinetex EVO C18 50 mm x 2.1 mm, 1.7 µm column (Phenomenex). UV-Vis chromatogram is acquired on a 200-400 nm range. Mass spectra are acquired in a 100-2000 Da range in positive mode with a 120000 resolution. ESI, *m/z* for [C_386_H_599_N_115_O_124_S_10_]^8+^ calculated m/z for A+1 ion: 1143.7658, measured: 1143.7662

**Figure S6: Repeatability of the oxidative folding the synthetic Schistosomin**

Monitoring of oxidative folding of the synthetic BgSmin precursor by analytical UPLC-MS starting from 2.8 mg (**A**) and 2.6 mg (**B**) of polypeptide. The unfolded linear precursors were first solubilized in 6 M guanidinium hydrochloride and diluted into a glutathione-based redox buffer supplemented with glycerol and the non-ionic detergent *n*-octylglucoside. Folding was carried out at 4°C and progressively converged over 32 days toward a single dominant species corresponding to the correctly folded BgSminSynth protein. The crudes were pooled and purified by RP-HPLC (0.85 mg, 17.4% isolated yield).

**A**

**B**

**Figure S7**. **Thermal denaturation profile of BgSmin^Synth^ monitored by Circular Dichroism.**

CD spectra were acquired on a Jasco J-815 spectropolarimeter equipped with a PFD-425S Peltier temperature control unit using BgSmin^Synth^ at 4 µM in 10 mM sodium phosphate buffer (pH 7.2). Ellipticity values (θ mdeg) at 222 nm were monitored as a function of temperature and converted in mean residue molar ellipticity [Θ]_MWR_.(deg.cm^2^.dmol^-1^ per residues).

**Figure S8. Molecular dynamics simulation.**

Root-mean-square fluctuation (RMSF in nm) comparison between BgSmin^A^ D88E backbone atoms **(A)** and BgSmin^P^ D88E backbone atoms **(B)**. Root mean square calculations were performed on backbone atoms. Data correspond to the mean ± SD (n = 3, 300 K).

**Figure S9. Schistosomin and conotoxin predicted structures.**

AlphaFold3-predicted structures of putative schistosomin-related peptides from various *Heterobranchia* gastropods and *Conus* peptides.

**Table S1. Pairwise structural similarity matrix based on Z-scores from the DALI server.**

Higher Z-scores (Holm et al., 2023) indicate greater structural similarity and reveal two main peptide clusters (Z-score from 6.8 to 7.4 in Yellow and 12.9 to 19.3 in orange), with lower similarity between groups indicating divergence in overall fold.

|  |  | **DALI Z-score** | | |  |  |  |  |  |  |  |  |  |  |  |  |  |
| --- | --- | --- | --- | --- | --- | --- | --- | --- | --- | --- | --- | --- | --- | --- | --- | --- | --- |
| **Source of peptide** | **1** | **2** | **3** | **4** | **5** | **6** | **7** | **8** | **9** | **10** | **11** | **12** | **13** | **14** | **15** | **16** | **17** |
| **1. *A. californica* Smin1** | 19.0 | 17.5 | 17.3 | 17.1 | 17.0 | 16.2 | 16.0 | 15.0 | 14.6 | 13.4 | 13.6 | 7.4 | 7.4 | 7.0 | 7.6 | 7.4 | 7.1 |
| **2. *A. californica* Smin2** | 17.5 | 18.9 | 17.1 | 17.0 | 16.7 | 16.2 | 16.0 | 14.8 | 14.5 | 13.3 | 13.7 | 7.3 | 7.4 | 7.1 | 7.5 | 7.3 | 7.1 |
| **3. *P. acuta* Smin1** | 17.3 | 17.1 | 18.9 | 17.8 | 17.0 | 16.1 | 15.9 | 15.0 | 14.5 | 13.2 | 13.7 | 7.4 | 7.6 | 7.1 | 7.7 | 7.4 | 7.1 |
| **4. *P. acuta* Smin2** | 17.1 | 17.0 | 17.8 | 18.9 | 16.8 | 16.0 | 15.7 | 15.0 | 14.5 | 13.3 | 13.9 | 7.4 | 7.6 | 7.2 | 7.7 | 7.5 | 7.2 |
| **5. *L. stagnalis*** | 17.0 | 16.7 | 17.0 | 16.8 | 19.0 | 16.2 | 15.8 | 14.5 | 14.6 | 13.0 | 13.5 | 7.5 | 7.6 | 7.2 | 7.7 | 7.4 | 7.2 |
| **6. *B. glabrata* Smin A** | 16.2 | 16.2 | 16.1 | 16.0 | 16.2 | 19.1 | 17.7 | 15.1 | 15.3 | 13.7 | 14.1 | 7.4 | 7.8 | 7.1 | 7.8 | 7.6 | 7.4 |
| **7. *B. glabrata* Smin B** | 16.0 | 16.0 | 15.9 | 15.7 | 15.8 | 17.7 | 19.2 | 14.9 | 15.4 | 13.8 | 14.0 | 7.6 | 7.7 | 7.2 | 7.7 | 7.6 | 7.1 |
| **8. *E. marginata*** | 15.0 | 14.8 | 15.0 | 15.0 | 14.5 | 15.1 | 14.9 | 19.3 | 13.8 | 14.3 | 14.8 | 7.4 | 7.4 | 6.9 | 7.5 | 7.2 | 6.9 |
| **9. *B. truncatus*** | 14.6 | 14.5 | 14.5 | 14.5 | 14.6 | 15.3 | 15.4 | 13.8 | 18.9 | 13.0 | 13.5 | 7.7 | 7.6 | 7.1 | 7.9 | 7.7 | 7.3 |
| **10. *E. chlorotica*** | 13.4 | 13.3 | 13.2 | 13.3 | 13.0 | 13.7 | 13.8 | 14.3 | 13.0 | 19.0 | 14.3 | 6.8 | 7.1 | 6.4 | 7.1 | 6.8 | 6.3 |
| **11. *P. ocellatus*** | 13.6 | 13.7 | 13.7 | 13.9 | 13.5 | 14.1 | 14.0 | 14.8 | 13.5 | 14.3 | 18.8 | 7.6 | 7.8 | 7.5 | 8.0 | 7.8 | 7.4 |
| **12. *C. geographus*** | 7.4 | 7.3 | 7.4 | 7.4 | 7.5 | 7.4 | 7.6 | 7.4 | 7.7 | 6.8 | 7.6 | 17.6 | 15.5 | 15.0 | 14.0 | 13.8 | 13.9 |
| **13. *C. litteratus*** | 7.4 | 7.4 | 7.6 | 7.6 | 7.6 | 7.8 | 7.7 | 7.4 | 7.6 | 7.1 | 7.8 | 15.5 | 17.5 | 15.3 | 13.9 | 13.8 | 13.2 |
| **14. *C. cactus*** | 7.0 | 7.1 | 7.1 | 7.2 | 7.2 | 7.1 | 7.2 | 6.9 | 7.1 | 6.4 | 7.5 | 15.0 | 15.3 | 17.4 | 13.2 | 13.3 | 12.9 |
| **15. *C. ebraeus*** | 7.6 | 7.5 | 7.7 | 7.7 | 7.7 | 7.8 | 7.7 | 7.5 | 7.9 | 7.1 | 8.0 | 14.0 | 13.9 | 13.2 | 17.0 | 13.3 | 14.0 |
| **16. *C. bayani*** | 7.4 | 7.3 | 7.4 | 7.5 | 7.4 | 7.6 | 7.6 | 7.2 | 7.7 | 6.8 | 7.8 | 13.8 | 13.8 | 13.3 | 13.3 | 17.2 | 12.9 |
| **17. *C. tulipa*** | 7.1 | 7.1 | 7.1 | 7.2 | 7.2 | 7.4 | 7.1 | 6.9 | 7.3 | 6.3 | 7.4 | 13.9 | 13.2 | 12.9 | 14.0 | 12.9 | 16.7 |

**Table S2. Primer sequences for qPCR analysis.**

| **Target** | **Forward (5’→3’)** | **Reverse (5’→3’)** |
| --- | --- | --- |
| Smine A | CTGCCAGGTTCTGTGTGTCTGG | GACAGGGATGCCGTTTGTTG |
| Smine P | CTGCCAGGTTCTGTGTGTCTGG | GGTTGTGTCTTGGTTGTGTCTTTAC |
| Actin | CAGGTTTCGCTGGAGACGAT | GCTGTCCTTCTGACCCATACCA |

**Supplementary information for Synthetic *B. glabrata* Schistosomin (BgSmin^Synth^) chemical synthesis referring to Figure S2, S3, S4 and S5**

Sequence of synthetic schistosomin:

DNYRCPNPGDAFECFESDATAR**F-C**VSGKRGAYVICSKCRRKYE**F-C**ANGAKVSKRPEVECRADWASTECTS**E**NSDVPSVMK(Biot)-NH_2_

Synthetic and biotinylated schistosomin is named BgSmin^Synth^ D88E with an alanine at position 78.

The junctions utilized for assembling the full-length protein and defining the peptide segments described hereinafter are highlighted in green. The protein is modified at the C-terminus by a lysine residue with a biotin group attached to its side-chain, to enable functional studies in the future. Note that the aspartic acid residue at position 88 (Uniprot numbering) has been substituted by a glutamic residue, since preliminary studies have revealed that D88-N89 junction is highly sensitive to aspartimide formation. The protein features a carboxamide group on its C-terminus.

**Reagents and solvents**

2-(1*H*-Benzotriazol-1-yl)-1,1,3,3-tetramethyluronium fluorophosphates (HBTU) and *N*-Fmoc protected amino acids were obtained from Iris Biotech GmbH.

3-mercaptopropionic acid (97%, MPA), 4-mercaptophenylacetic acid (97%, MPAA), *tris*(2-carboxyethyl)phosphine hydrochloride (98%, TCEP∙HCl), guanidine hydrochloride (Gn∙HCl, 99%), triisopropylsilane (TIS), thiophenol, thioanisole, ethane-1,2-dithiol (EDT), hydrochloric acid (reagent grade, 37% w/v), glacial acetic acid (AcOH), acetic anhydride (Ac_2_O), trifluoroacetic acid (TFA), sodium hydroxide (pellets, 97%), piperidine, *N,N,N*-diisopropylethylamine (DIEA), triethylamine (TEA, as well as other reagents were purchased from suppliers of chemicals (TCI, Acros Organics, Sigma-Aldrich, Merck…) and were of the purest grade available.

Peptide synthesis grade *N*,*N*-dimethylformamide (DMF), dichloromethane (DCM), 1,2-dichloroethane (DCE), acetone, methanol (MeOH), ethanol (EtOH), diethyl ether (Et_2_O), acetonitrile, heptane, LC–MS-grade acetonitrile (0.1% TFA), LC–MS grade water (0.1% TFA) and trifluoroacetic acid (TFA) were purchased from Biosolve and Fisher-Chemical. Water was purified with a Milli-Q Ultra Pure Water Purification System.

Bis(2-sulfenylethyl)amino trityl 1% divinylbenzene crosslinked polystyrene solid support (SEA solid support) was produced on demand by Roowin.

Solvents and reagents were used as received.

**Caution:**

TFA is an extremely corrosive liquid; great care must be taken when using this reagent. Proper eye protection, lab coat, and gloves are mandatory. TFA must be manipulated under a fume hood.

DMF is a highly toxic solvent. Proper eye protection, lab coat, and gloves are mandatory. DMF must be manipulated under a fume hood.

**Instrumentation**

The following instrumentation was used to synthesize, purify and characterize the peptides prepared in this work.

Unless otherwise stated, peptides were synthesized using an automated peptide synthesizer (Intavis) and standard Fmoc solid phase peptide synthesis methods.

Reaction mixtures or isolated compounds were characterized by analytical UPLC–MS using a System Ultimate 3000 UPLC (Thermofisher) equipped with a C18 column, a diode array detector and a mass spectrometer (Ion trap LCQfleet, heat temperature 350 °C, spray voltage 2.8 kV, capillary temperature 350 °C, capillary voltage 10 V, tube lens voltage 75 V). Unless otherwise stated, analyses were performed using an appropriate linear gradient of eluent B (0.1% TFA in CH_3_CN) in eluent A (0.1% TFA in H_2_O) at a flow rate of 0.4 mL/min.

Peptides were purified by preparative RP-HPLC using a Buchi system equipped with C18 column and an UV detector. Purifications were performed using an appropriate linear gradient of eluent B (0.1% TFA in CH_3_CN) in eluent A (0.1% TFA in H_2_O) at a flow rate of 6 mL/min. Selected fractions were combined, frozen and lyophilized.

**General protocols for peptide synthesis**

*General protocol for peptide elongation*

Peptide elongation was performed using standard Fmoc/*tert*-butyl chemistry on an automated peptide synthesizer. Couplings were performed using 5-fold molar excess of each Fmoc-L-amino acid, 4.5-fold molar excess of TBTU, and 10-fold molar excess of DIEA. A capping step was performed after each coupling with Ac_2_O/DIEA in DMF. At the end of the synthesis, the peptidyl solid support was washed with CH_2_Cl_2_, diethyl ether and dried *in vacuo*.

Final peptide deprotection and cleavage: The elongated peptide was cleaved from the solid support using TFA with appropriate scavengers as indicated in each case. The crude peptide was precipitated by adding the TFA solution coming from the cleavage and deprotection step in ice-cold diethyl ether/heptane 1/1 v/v (20 mL per mL of TFA cocktail) and recovered by centrifugation. The solid was washed with cold diethyl ether/heptane 1/1 v/v (40 mL), dissolved in water and the solution was lyophilized.

*Peptide amide* ***5***

Peptides amides were synthesized using standard Fmoc solid phase peptide synthesis methods on a NovaSyn TGR solid support (0.25 mmol/g).

CANGAKVSKRPEVECRADWASTECTSENSDVPSVMK(Biot)-NH_2_ **5**, 0.10 mmole scale; Cleavage cocktail: TFA/water/EDT/TIS 90/2.5/2.5/5 by vol; Yield: 56.8 mg (12.4%).

*SEA^off^ peptide segments*

Synthesis of SEA^off^ peptide segments was performed on SEA PS resin (0.16 mmol/g) using standard Fmoc/*tert*-butyl chemistry on an automated peptide synthesizer. Typical procedures for the synthesis of SEA^off^ peptide segments were described in detail in previous papers. For detailed protocols see references (1, 2). The synthesis requires that all Cys residues are temporarily protected by a tert-butylsulfenyl (S*t*Bu) group, that remains on the peptide following the deprotection and cleavage step in TFA.

DNYRC(S*t*Bu)PNPGDAFEC(S*t*Bu)FESDATARF-SEA^off^ **I** was prepared as described elsewhere (0.1 mmole scale).(3) Cleavage cocktail: TFA/water/thioanisole/thiophenol//TIS 87.5/2.5/2.5/2.5/5 by vol; Yield: 47.3 mg (14.5%)

C(S*t*Bu)VSGKRGAYVIC(S*t*Bu)SKC(S*t*Bu)RRKYEF-SEA^off^ **2** was prepared as described elsewhere (0.1 mmole scale).(3) Cleavage cocktail: TFA/water/thioanisole/thiophenol//TIS 87.5/2.5/2.5/2.5/5 by vol; Yield: 42.5 mg (12%)

*MPA peptide thioester* ***1***

Peptide thioester **1** was prepared by exchanging the SEA group of SEA peptide **I** by 3-mercaptopropionic acid (MPA) at pH 4.0 according to published procedures (2, 3).

The exchange has been performed on 22.3 mg (6.60 µmoles) of SEA^off^ peptide **I**. The reaction provided 8.5 mg (41 %) of peptide thioester **1** after HPLC purification.

**References:**

1. Ollivier N, Dheur J, Mhidia R, Blanpain A, Melnyk O. Bis(2-sulfanylethyl)amino native peptide ligation. Org Lett. 2010;12(22):5238-41.

2. Ollivier N, Raibaut L, Blanpain A, Desmet R, Dheur J, Mhidia R, et al. Tidbits for the synthesis of bis(2-sulfanylethyl)amido (SEA) polystyrene resin, SEA peptides and peptide thioesters. J Pept Sci. 2014;20(2):92-7.

3. Dheur J, Ollivier N, Vallin A, Melnyk O. Synthesis of peptide alkylthioesters using the intramolecular N,S-acyl shift properties of bis(2-sulfanylethyl)amido peptides. J Org Chem. 2011;76(9):3194-202.
